# Association between usage of household cooking fuel and congenital birth defects-18 months multi-centric cohort study in Nepal

**DOI:** 10.1186/s13690-023-01169-1

**Published:** 2023-08-11

**Authors:** Ashish KC, Sanni Halme, Rejina Gurung, Omkar Basnet, Erik Olsson, Ebba Malmqvist

**Affiliations:** 1 School of Public Health and Community Medicine, University of Gothenburg, Medicinargatan 18, Gothenburg, Sweden; 2 Department of Women’s and Children’s Health, Uppsala University, Uppsala, Sweden; 3 Research Division, Golden Community, Lalitpur, Nepal; 4 Lund University, Biskopsgatan 9, Lund, Sweden

**Keywords:** Air ventilation, Congenital birth defect, Indoor environment, Neonatal health, Polluted cooking fuel

## Abstract

**Background:**

- An estimated 240,000 newborns die worldwide within 28 days of birth every year due to congenital birth defect. Exposure to poor indoor environment contributes to poor health outcomes. In this research, we aim to evaluate the association between the usage of different type household cooking fuel and congenital birth defects in Nepal, as well as investigate whether air ventilation usage had a modifying effect on the possible association.

**Methods:**

- This is a secondary analysis of multi-centric prospective cohort study evaluating Quality Improvement Project in 12 public referral hospitals of Nepal from 2017 to 2018. The study sample was 66,713 women with a newborn, whose information was available in hospital records and exit interviews. The association between cooking fuel type usage and congenital birth defects was investigated with adjusted multivariable logistic regression. To investigate the air ventilation usage, a stratified multivariable logistic regression analysis was performed.

**Results:**

-In the study population (N = 66,713), 60.0% used polluting fuels for cooking and 89.6% did not have proper air ventilation. The prevalence rate of congenital birth defect was higher among the families who used polluting fuels for cooking than those who used cleaner fuels (5.5/1000 vs. 3.5/1000, p < 0.001). Families using polluting fuels had higher odds (aOR 1.49; 95% CI; 1.16, 1.91) of having a child with a congenital birth defect compared to mothers using cleaner fuels adjusted with all available co-variates. Families not using ventilation while cooking had even higher but statistically insignificant odds of having a child with congenital birth defects (aOR 1.34; 95% CI; 0.86, 2.07) adjusted with all other variates.

**Conclusion:**

- The usage of polluted fuels for cooking has an increased odds of congenital birth defects with no significant association with ventilation. This study adds to the increasing knowledge on the adverse effect of polluting fuels for cooking and the need for action to reduce this exposure.

**Supplementary Information:**

The online version contains supplementary material available at 10.1186/s13690-023-01169-1.

**Table:** 

**Text box 1. Contributions to the literature**
• Congenital birth defect is one of the global burdens which can cause infant death as well as disability later in life. Understanding of the environmental factors attributing to congenital birth defect will help to develop intervention to reduce the risk.
• In Nepal, two third of the household use polluted fuel for cooking and four out of five household donot have ventilation in the kitchen. This study showed that use of polluted fuel increases the odds of congenital birth defect by 49% in comparison to household where the clean fuel is used for cooking.

## Introduction

Birth defects increase the risk of neonatal and childhood mortality and disability [1–3]. Annually estimated 240,000 newborn die globally due to birth defects during the critical first 28 days of birth, whereas additional 170,000 deaths occur among children aged one to five years [1–4], mostly in low- and middle-income countries. There are different factors that exacerbate the risk of congenital birth defect such as lack of access to healthcare services, inadequate access to nutrition-rich food, high rates of infection and a high exposure to some environmental factors [5–11].

Globally, an estimation of 2.6 billion people donot practice or have access to safe and clean cooking practices [12–15]. Many households, especially in low- and middle-income countries, are using simple stoves and solid fuels as the energy and cooking fuel of their household [16]. The inefficient and incomplete combustion of solid fuel (SF) is associated with high levels of indoor air pollutants leading to 3.55 million deaths annually with higher risk to women and children [17]. Moreover, reduction of the household air pollution also requires focus on proper air ventilation in the kitchen [15, 18]. Air ventilation can reduce greatly the effect of polluted cooking fuel on health hazards caused by the household air pollution [12, 17]. The challenge is that many households are cooking with simple cooking stoves, and they lack air ventilation in the kitchen, which causes higher number of polluted emissions [16, 19].

Maternal exposure to household air pollution can increase the risk for adverse pregnancy and childbirth outcomes: stillbirths and low birth weight [20]. Environmental exposures, including air pollution, are risk factors for birth defects [21]. A recent study conducted in China, found a significant association between birth defects and polluted cooking fuel usage: the risk of any birth defect is higher if the household uses polluted cooking fuel, such as plant residue or firewood, compared to households using gas [22]. Moreover, a study conducted in several low-income countries revealed, that children, whose mother was exposed to smoke from an open fire while cooking during the pregnancy were at higher risk of orofacial birth defects [23, 24]. A critical review found that both, indoor and ambient air pollution are related to the increased risk of some birth defects, especially NTDs, oral clefts, and heart defects; however, there are few studies regarding indoor air pollution [21, 24].

Furthermore, passive or maternal smoking contributes to the household air pollution and fetal exposure during pregnancy and therefore, can highly increase the risk of some birth defects [21, 24]. Moreover, the connection between birth defects and cooking fuel usage at the household is understudied in different contexts and it is generally not well established [22].

The aim of this research is to study the association between household cooking fuel type usage and congenital birth defects in Nepal and investigate whether ventilation usage in the kitchen while cooking moderates this possible association.

## Methods

### Study design

This study is a secondary analysis of a prospective cohort study for Nepal Perinatal Quality Improvement Project (NePeriQIP)[25].

### Study setting

The primary study was conducted in 12 different referral hospitals in Nepal to evaluate the effect of quality improvement package on perinatal outcomes. Of the public referral hospitals of the country, hospitals which had an annual birth of 1000 were selected. All these hospitals had comprehensive emergency obstetric and newborn care (CEONC) set up for managing complicated birth and special newborn care unit for managing sick newborns. In special newborn care units, sick newborns both outborn’s and inborn’s were admitted with standard operating protocol for managing sick newborns. The standard clinical definition of congenital birth defect in these hospitals was any structural anomalies that occur during intrauterine period (before birth) and identified during the clinical examination after birth (immediately after birth and before discharge)[26, 27].

### Participants inclusion criteria

Women admitted in the study hospitals with the gestational age ≥ 22 weeks for childbirth with fetal heart sound at admission were eligible for the primary study. Women who consented to the primary study with information extracted on clinical evaluation of congenital birth defect and interview with women at discharge were eligible for this study. Exclusion criteria was woman with antepartum stillbirth (no signs of life before labour) and no fetal heartbeat sound during the arrival to labor ward.

Data collection- For the primary study, an independent data collection team was established in the 12 hospitals. The data collectors assessed the eligibility of women for enrollment and consented eligible participants to the study. The data collector followed each woman from the time of admission until discharge and extracted the demographic, obstetric and neonatal clinical information from maternity register and individual patient record book in a paper form. During the record extraction, all newborn care information including congenital birth defect was extracted. At the time of discharge, each woman was interviewed on their socio-economic status, housing conditions, use fuel for cooking and kitchen ventilation in the house. The data extraction tool and interview questionnaire were pre-tested for their reliability before the start of the study. The data collection was done between the April 2017 and October 2018 in all 12 hospitals at the same time.

### Measurement

*Exposure -* The cleaner fuel included electricity, liquid petroleum gas (LPG), biogas, and natural gas usage. The polluting fuel include firewood and kerosene usage. The variable was collected through interview with woman.

*Outcome-*The studied outcome variable was newborns with a diagnosed structural anomalies occurring during intrauterine period detected on clinical examination immediately after birth and at the time of discharge. The variable information was collected through clinical record extraction.

Other covariates.

- Maternal educational level was categorized in three groups: no education, primary education (5 years of formal education) and, secondary (10 years of formal education) and higher. The variable was collected through interview with woman.
- Ethnicity was defined as two categorical groups: advantaged and disadvantaged. Advantaged group included Brahmin/Chhetri, since they are classified as higher class in Nepal. Similarly, disadvantaged group included Dalit, Madhesi, Muslim, Janajati and others, since they are seen as a lower class in Nepal [28]. The variable information was collected through interview with woman.
- Household monthly income [29]- We used household monthly income in the study instead of household wealth. This was because the wealth index was constructed including firewood usage (combined with income and Liquid Petroleum Gas). The exposure would thus be partly the same as the outcome. The monthly incomes were divided into four groups, quartile based on the frequencies of the incomes. The lowest quantile has income less than 500 USD, third quartile has income between 50 and 100 USD, second quartile has income between 100 and 250 USD and first quartile has income between 250 and 1800 USD. The variable information was collected through interview with woman.
- Sex of the child categories as male and female. The variable information was collected through clinical record extraction.
- Parity defined as nulliparous as 0 previous birth, primiparous as 1 previous birth and multiparous as two or more previous births. The variable information was collected through clinical record extraction.
- The access to kitchen ventilation defined as kitchen having chimney, hood or either. The variable information was collected through interview with woman.

### Statistical methods

Bivariate analysis was performed to assess the crude associations between all the included variables and birth defects with outcome of interest.

The chosen confounders for adjusting were maternal age; maternal education; ethnicity; and household monthly income. Factors affecting to congenital birth defects were included in the study as variance reduction: sex of the child; parity; and single -or multiple pregnancy. Firstly, unadjusted logistic regression was performed between the exposure and outcome. Thereafter, adjusted logistic regressions were performed to control for the confounding factors that are related to both to the exposure and the outcome according to existing literature [22, 30, 31]. Adjusted logistic regression was conducted in Model 1, which was adjusted only for confounders. Adjusted Model 2 was adjusted for confounders and also for the variables associated only with the outcome. This adjustment was done to reduce variance in the outcome and increase statistical power. Hence, Adjusted Model 2 was considered as the main model of this study. The reference categories in the regression analyses were: clean fuel usage, no birth defect, < 20 years, no education, advantaged ethnicity, lowest income, males, no previous pregnancies, and single pregnancy.

Separate stratified multivariable regression analysis with air ventilation usage (yes and no) was conducted to see whether air ventilation is an effect modifier of the association. The stratified analysis was conducted among participants not using air ventilation while cooking (N = 59,806) and separately among participants using air ventilation (N = 6,907). Both unadjusted and adjusted models were conducted. In the adjusted model of participants not using air ventilation, birth defects were adjusted for exposure (cooking fuel type usage) and all covariates (maternal age, maternal education, ethnicity, household monthly income, sex of the child, parity and single or multiple delivery). Whereas, in adjusted model of participants using air ventilation, birth defects and cooking fuel type usage were adjusted for covariates (maternal age, maternal education, ethnicity, household monthly income sex of the child, and parity). The estimates between the covariates are not interpreted in this study. The specific covariates were selected based on existing literature, in order to avoid the impact of multicollinearity.

## Result

**Fig. 1:**
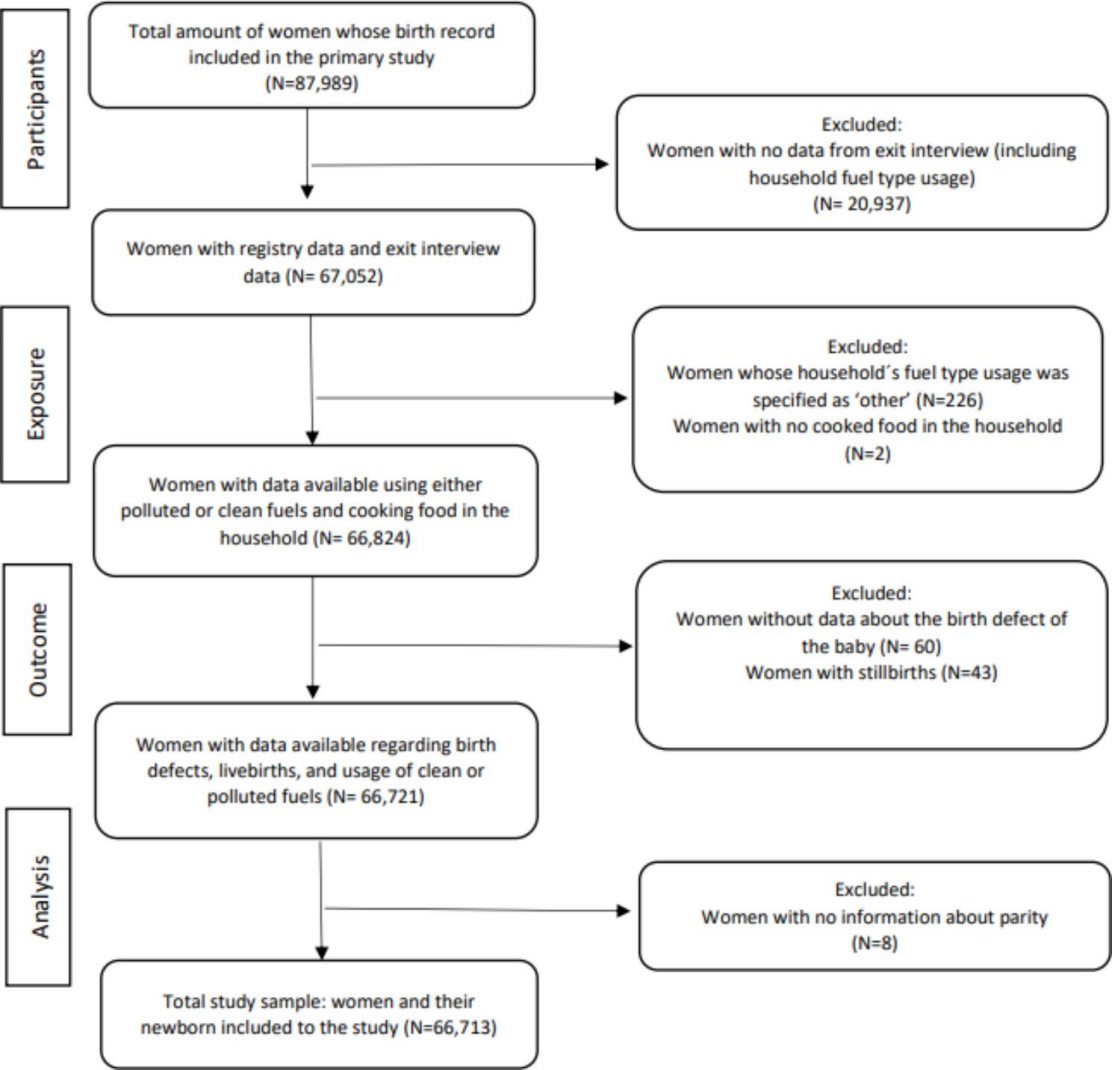
Flow chart of the study participants 2017–2018 in Nepal

The total number of women enrolled in the study was 87,989, among whom 67,052 participated in the pre-discharge exit interview with 66,713 women-newborn pair with all required data available. (Fig. 1) In the study sample, 40.0% of the households were using clean fuels for cooking (electricity, LPG, biogas or natural gas), whereas 60.0% were using polluted fuel (kerosene or firewood). Notably, only 10.4% of the study sample had access to air ventilation The prevalence of birth defect in the sample was 0.5% (N = 313). (Table 1)

**Table 1:** Background characteristics of the participants 2017–2018 in Nepal

Characteristic	Frequency N (%) (N = 66,713)
**Household fuel usage** (exposure)	
Cleaner fuel (electricity, LPG, biogas, natural gas)	26,703 (40.0)
Polluting fuel (kerosene, firewood)	40,010 (60.0)
**Air ventilation of the cooking place**	
No	59,806 (89.6)
Yes	6,907 (10.4)
**Congential Birth defect** (outcome)	
No	66,400 (99.5)
Yes	313 (0.5)

The prevalence rate of congenital birth defect was higher among families who used polluted fuel for cooking than those who used cleaner fuel (5.5/1000 vs. 3.5/1000, p < 0.001). The prevalence of congenital birth defect was higher among families who had no ventilation in the kitchen than those who had ventilation but statistically insignificant (4.9/1000 vs. 3.2/1000, p = 0.056). The prevalence rate of congenital birth defect was higher among the women less than 20 years (6.9/1000) and those age 35 years or above (6.7/1000 compared to 20–34 years old (3.8/1000) (p = 0.031). The prevalence rate was higher among the relatively disadvantaged ethnicities than the relatively advantaged ethnicity (5.7/1000 vs. 3.1/1000, p < 0.001). The prevalence rate was higher among the household with lowest income than those with highest income (6.7/1000 vs. 2.7/1000, p < 0.001). The prevalence rate was higher among female newborns than male infants (5.6/1000 vs. 3.9/1000, p = 0.001). The prevalence rate was higher among newborns born to multiparous women than those born to nulliparous women (6.7/1000 vs. 3.9/1000, p < 0.001). Lastly, the prevalence rate was also higher among singleton born than multiple born newborns (13.4/1000 vs. 4.6/1000, p = 0.002). (Table 2)

**Table 2:** Prevalence rate of congenital birth defect by exposure (cooking fuel type usage) and covariates 2017–2018 in Nepal

Variables	Birth defect among the sub-group	Birth defect per 1000 live born	P-value
**Cooking fuel type usage**			
Cleaner fuel usage	93/26,610	3.5/1000	< 0.001
Polluting fuel usage	220/39,790	5.5/1000	
**Indoor ventilation**			
Yes	22/6907	3.2/1000	0.056
No	291/59,806	4.9/1000	
**Maternal age**			
< 20	35/5,039	6.9/1000	0.031
20–26	213/45,664	4.7/1000	
27–34	54/14,049	3.8/1000	
35 or over	11/1648	6.7/1000	
**Maternal education**			
No education	39/9,421	4.1/1000	0.573
Basic education	49/10,984	4.5/1000	
Secondary and higher	225/45,995	4.9/1000	
**Ethnicity**			
Advantaged	80/25,503	3.1/1000	< 0.001
Disadvantaged	233/40,897	5.7/1000	
**Household monthly income**			
Lowest	189/28,307	6.7/1000	< 0.001
Lower	23/5,275	4.4/1000	
Higher	58/17,120	3.4/1000	
Highest	43/15,698	2.7/1000	
**Sex of the child**			
Male	141/36,059	3.9/1000	0.001
Female	172/30,541	5.6/1000	
**Parity**			
0 previous births	125/32,228	3.9/1000	< 0.001
1 previous birth	112/22,807	4.9/1000	
2 or more previous births	76/11,365	6.7/1000	
**Single or multiple pregnancy**			
Single	305/65,801	4.6/1000	0.002
Multiple	8/599	13.4/1000	

**Fig. 2:**
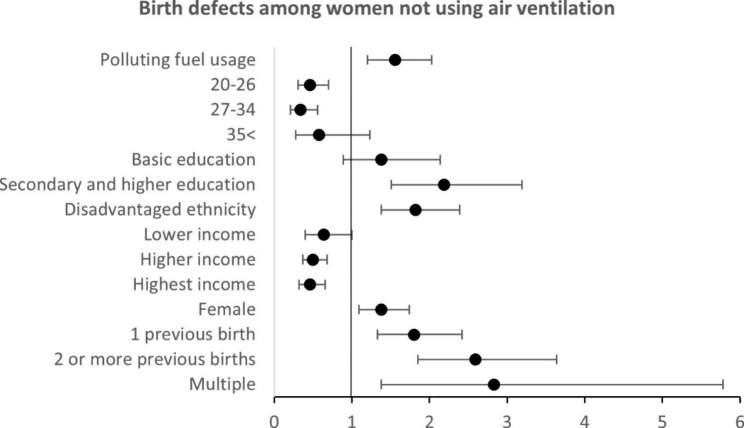
Forest plot on the adjusted odd ratio to congential birth defect for those exposed to indoor pollution with no indoor air ventilation 2017–2018 in Nepal

In the unadjusted analysis, participants not using ventilation and used polluted fuel for cooking, had 1.63 higher odds of having a child with birth defect (cOR, 1.63; 95% CI; 1.27, 2.11, p-value: <0.001), compared to the women using cleaner fuel. (Fig. 2, supplementary Table 2). The crude odds ratio showed a significant association between the household type of cooking fuel and congenital birth defect. Women who were using polluting fuel for cooking had around 1.58 times higher odds of having a child with birth defect (OR, 1.58; 95% CI; 1.24, 2.02) compared to the women who used cleaner cooking fuel. The crude odds ratio showed an insignificant association between ventilation and congenital birth defect (OR, 1.34; 95% CI; 0.86, 2.07). After adjusting with ventilation, maternal age, maternal education, ethnicity, income, sex and parity, women using polluting fuel were at 1.49 times higher odds to have a child with birth defect (aOR, 1.49; 95% CI; 1.16, 1.91), compared to women using cleaner fuel (Table 3).

**Table 3:** Association between outcome (congenital birth defect), exposure (household cooking fuel type usage) and all covariates 2017–2018 in Nepal

	Unadjusted		Adjusted Model	
	OR (95% CI)	P-value	aOR (95% CI)	P-value
**Household cooking fuel type usage**				
Cleaner fuel	Ref		Ref	
Polluting fuel	1.58 (1.24–2.02)	< 0.001	1.49 (1.16–1.91)	0.002
**Air ventilation**				
**Yes**	Reference			
**No**	1.53 (0.99–2.36)	0.055	1.34 (0.86–2.07)	0.195
**Maternal age**				
< 20	Ref		Ref	
20–26	0.67 (0.47–0.96)	0.030	0.51 (0.34–0.75)	< 0.001
27–34	0.55 (0.36–0.85)	0.007	0.41 (0.25–0.65)	< 0.001
35<	0.96 (0.49–1.90)	0.909	0.60 (0.29–1.25)	0.170
**Maternal education**				
No education	Ref		Ref	
Basic education	1.08 (0.71–1.64)	0.728	1.28 (0.84–1.96)	0.249
Secondary and higher	1.18 (0.84–1.66)	0.337	2.00 (1.40–2.86)	< 0.001
**Ethnicity**				
Advantaged	Ref		Ref	
Disadvantaged	1.82 (1.41–2.34)	< 0.001	1.81 (1.39–2.35)	< 0.001
**Household monthly income**				
Lowest	Ref		Ref	
Lower	0.65 (0.42–1.01)	0.054	0.64 (0.42-1.00)	0.042
Higher	0.51 (0.38–0.68)	< 0.001	0.53 (0.39–0.71)	< 0.001
Highest	0.41 (0.29–0.57)	< 0.001	0.49 (0.35–0.68)	< 0.001
**Sex of the child**				
Male	Ref		Ref	
Female	1.45 (1.16–1.81)	0.001	1.47 (1.17–1.83)	< 0.001
**Parity**				
0 previous births	Ref		Ref	
1 previous birth	1.27 (0.98–1.64)	0.070	1.64 (1.24–2.18)	< 0.001
2 or more previous births	1.72 (1.30–2.30)	< 0.001	2.30 (1.66–3.20)	< 0.001
**Single or multiple pregnancy**				
Single	Ref		Ref	
Multiple	2.88 (1.42–5.84)	0.003	2.70 (1.32–5.52)	0.007

Adjusted for clean vs. polluted fuel, ventilation, age stratified, maternal education, Ethnicity, income per month, sex of the newborn, parity and multiple delivery

In the stratified analyses, participants not using air ventilation, 36 000 (60.2%) were using polluting fuel and 23 806 (39.8%) were using cleaner fuel. Whereas among participant using air ventilation, 4010 (58.1%) were using polluting fuel and 2897 (41.9%) were using cleaner fuel (supplementary Table 1).

## Discussion

Polluted cooking fuel and congenital birth defects are significantly associated in the adjusted model when controlling for ventilation, maternal age, maternal education, ethnicity, household monthly income, sex of the child, parity and single or multiple delivery. Mothers using polluted fuel were more likely to have a child with congenital birth defect compared to the mothers using clean fuel for cooking. Furthermore, the air ventilation usage while cooking did modify the association between cooking fuel type usage and congenital birth defects: women cooking with polluted fuels and not using air ventilation were more likely to have a child with birth defects compared to those using clean fuels. Additionally, the association between cooking fuel type usage and birth defects was not significant among women using air ventilation.

This study suggests that usage of polluted fuels is associated with birth defects by increasing the odds compared to the usage of clean fuels. The mechanisms behind the formation of birth defects are relatively unknown, even though some risks factors have been recognized [32]. Additionally, the factors behind the association between cooking fuel type usage and birth defects is under studied and inadequately established [33]. Previous research suggests biological mechanism behind this association between the pregnant mother and the fetus [34]. The small particles from ambient air pollutant emissions, such as PM, PAHs and NO_2,_ have found to be able to pass the placental barrier [34]. When inhaling the polluted air, small particles are able to pass to the blood circulation of the mother ending up passing the placental barrier [34]. Possible reasons for air pollution emission causing birth defects may be epigenetic changes, oxidative stress and placental inflammation [34]. Animal tests have also shown, that placental inflammation may disturb the oxygen and nutrient flow through the placenta for the fetus, which may lead to insufficient growth, birth defects and even stillbirths [35].

The present study found significant association between polluted cooking fuel usage and birth defects in Nepal. The results are in alignment with previous research conducted in China, where the association between polluted fuel usage and birth defects were significantly associated and were more likely to occur if mothers were exposed to polluted cooking fuels during pregnancy compared to mothers using clean fuels [22]. A recent study by Sun et al. found out that exposure to high levels of indoor pollutants can increase the risk of heart defects [36]. This suggests that the exposure level can affect the outcome. Several things can affect to the level of exposure: the level of pollutant particles in household air combusted from polluted fuels; duration spent for cooking; general duration spent indoors; how often cooked indoors per day; and usage of air ventilation [12, 37, 38].

However, this study included air ventilation as an important aspect in the reduction of the exposure. This study found strong association between the usage of polluted fuels and increased odds on having a child with birth defects among women who did not use air ventilation. However, the association lost its significance when studying the association among women using air ventilation while cooking. These results may reflect that polluted fuel is associated with increased odds of birth defects mainly when there is no usage of air ventilation while cooking.

Although air ventilation usage might have a decreasing effect on the level of household air pollution and therefore decreased risk of birth defects, also kitchen location plays a role in the level of exposure [15]. Cooking in a kitchen at separate room, especially when cooking with polluted fuels, is important in order to reduce its negative impacts on health [39]. In Nepal, households are more likely to have separate kitchen when having higher SES and with lower SES it is more common not to have separate room for kitchen [39]. However, this study did not include data regarding separate room for kitchen, which could have made difference on the association between cooking fuel type usage and birth defects and especially on the modifying effect of air ventilation.

### Methodological consideration

The modification of the association between cooking fuel type usage and birth defects by air ventilation is limited as the women using air ventilation was much fewer than women not using air ventilation. Due to the smaller sample size, some response categories in the covariates were combined in order to get more participants to each category. Nevertheless, the sample size of women using air ventilation remained relatively high in total. The type of congenital birth defect was not recorded, so this study does not provide the risk type by exposure to polluted fuel.

## Conclusion

Reducing the usage of polluted fuel and improving ventilation can reduce the risk of birth defect. Improvement in generation and consumption of clean energy fuel will be a future endeavor for Nepal. Improving societal knowledge for a well-ventilated kitchen and usage of clean fuel for indoor cooking is important for the better health and survival of people residing in household as well as for the unborn infant. The Sustainable Development Goal 13 have recognized and addressed the need for sustainable, affordable, and healthy energy usage not only for improving human health but for a healthier planet.

### Electronic supplementary material

Below is the link to the electronic supplementary material.

Supplementary Material 1: **Table 1.** Distribution of women who have ventilation with type of fuel used for cooking 2017–2018 in Nepal. **Table 2.** Stratified analysis including the participants not using air ventilation while cooking (N = 59,806) on the association between household cooking fuel type usage and birth defects 2017–2018 in Nepal

## Data Availability

The datasets generated and/or analyzed during the current study are not publicly available due to the involvement of information of individuals disclosing identifiers but are available from the corresponding author on reasonable request.

## Abbreviations

OR: Odds ratio
aOR: Adjusted odds ratio
CI: Confidence interval
NePeriQIP: Nepal Perinatal Quality Improvement Project
SDG: Sustainable Development Goal
SES: Socio-Economic Status
CEONC: Comprehensive Emergency Obstetric and Newborn Care
LPG: Liquid Petroleum Gas
