# Supplementary material for: Association between usage of household cooking fuel and congenital birth defects-18 months multi-centric cohort study in Nepal
Source: Arch Public Health. 2023 Aug 11;81:144. doi: 10.1186/s13690-023-01169-1 (PMC10416396; doi:10.1186/s13690-023-01169-1)
Supplement: Supplementary file 1 — Supplementary Material 1: Table 1. Distribution of women who have ventilation with type of fuel used for cooking 2017–2018 in Nepal. Table 2. Stratified analysis including the participants not using air ventilation while cooking (N = 59,806) on the association between household cooking fuel type usage and birth defects 2017–2018 in Nepal [file 13690_2023_1169_MOESM1_ESM.docx]

*Supplementary table 1. Distribution of women who have ventilation with type of fuel used for cooking* 2017-2018 in Nepal

|  | Use of clean fuel n=26,703 (40.0%) | Use of polluted fuel n=40,010 (60.0%) |
| --- | --- | --- |
| Kitchen with no ventilation (59,806) | 23,806 (39.8%) | 36,000 (60.2%) |
| Kitchen with ventilation (6,907) | 2,897 (41.9%) | 4,010 (58.1%) |

*Supplementary table 2: Stratified analysis including the participants not using air ventilation while cooking (N=59,806) on the association between household cooking fuel type usage and birth defects* 2017-2018 in Nepal

|  | **Crude** |  | | **Adjusted Model** | |  | |  |
| --- | --- | --- | --- | --- | --- | --- | --- | --- |
|  | OR (95% CI) | P-value | | aOR (95% CI) | | P-value | |  |
| **Household cooking fuel usage** |  |  | |  | |  | |  |
| Cleaner fuel | Ref |  | | Ref | |  | |  |
| Polluting fuel | 1.63 (1.27-2.11) | <0.001 | | 1.56 (1.20-2.03) | | <0.001 | |  |
| **Maternal age** |  |  | |  | |  | |  |
| <20 | Ref |  | | Ref | |  | |  |
| 20-26 | 0.65 (0.45-0.95) | 0.024 | | 0.46 (0.31-0.70) | | <0.001 | |  |
| 27-34 | 0.54 (0.35-0.84) | 0.006 | | 0.34 (0.21-0.56) | | <0.001 | |  |
| 35< | 1.03 (0.52-2.04) | 0.940 | | 0.58 (0.28-1.23) | | 0.156 | |  |
| **Maternal education** |  |  | |  | |  | |  |
| No education | Ref |  | | Ref | |  | |  |
| Basic education | 1.11 (0.72-1.72) | 0.638 | | 1.38 (0.89-2.14) | | 0.156 | |  |
| Secondary and higher | 1.23 (0.86-1.75) | 0.261 | | 2.19 (1.51-3.19) | | <0.001 | |  |
| **Ethnicity** |  |  | |  | |  | |  |
| Advantaged | Ref |  | | Ref | |  | |  |
| Disadvantaged | 1.80 (1.38-2.35) | <0.001 | | 1.82 (1.38-2.39) | | <0.001 | |  |
| **Household monthly income** | | |  | |  | |  | |
| Lowest | Ref |  | | Ref | |  | |  |
| Lower | 0.65 (0.41-1.02) | 0.061 | | 0.64 (0.40-1.00) | | 0.052 | |  |
| Higher | 0.48 (0.35-0.65) | <0.001 | | 0.50 (0.37-0.68) | | <0.001 | |  |
| Highest | 0.39 (0.28-0.56) | <0.001 | | 0.46 (0.32-0.66) | | <0.001 | |  |
| **Sex of the child** |  |  | |  | |  | |  |
| Male | Ref |  | | Ref | |  | |  |
| Female | 1.36 (1.08-1.72) | 0.009 | | 1.38 (1.09-1.74) | | 0.007 | |  |
| **Parity** |  |  | |  | |  | |  |
| 0 previous births | Ref |  | | Ref | |  | |  |
| 1 previous birth | 1.34 (1.02-1.75) | 0.034 | | 1.80 (1.33-2.42) | | <0.001 | |  |
| 2 or more previous births | 1.90 (1.41-2.54) | <0.001 | | 2.59 (1.85-3.64) | | <0.001 | |  |
| **Single or multiple pregnancy** |  |  | |  | |  | |  |
| Single | Ref |  | | Ref | |  | |  |
| Multiple | 3.11 (1.53-6.31) | 0.002 | | 2.83 (1.38-5.78) | | 0.004 | |  |

OR= Odds ratio, aOR= Adjusted odds ratio, CI= Confidence interval
